# Mesenchymal Stem Cells Alleviate DHEA-Induced Polycystic Ovary Syndrome (PCOS) by Inhibiting Inflammation in Mice

**DOI:** 10.1155/2019/9782373

**Published:** 2019-09-12

**Authors:** Qi Xie, XingLiang Xiong, Na Xiao, Ke He, Maosheng Chen, Jing Peng, Xian Su, Hua Mei, Yanni Dai, Dan Wei, Ge Lin, Lamei Cheng

**Affiliations:** ^1^Institute of Reproduction and Stem Cell Engineering, School of Basic Medical Science, Central South University, Changsha 86 Hunan, China; ^2^National Engineering Research Centre of Human Stem Cells, Changsha 86, China; ^3^Reproductive and Genetic Hospital of CITIC-Xiangya, Changsha 86, China

## Abstract

Polycystic ovary syndrome (PCOS) is the most common cause of anovulatory infertility in women of reproductive age. Chronic inflammation is considered to be the cause of ovarian dysfunction. Increasing evidence in animal studies and in preliminary clinical trials has demonstrated that MSCs possess immunomodulatory effects via their interaction with immune cells. However, their contribution to PCOS remains unclear. In this study, we showed that the administration of hUC-MSCs could efficiently improve the pathological changes of PCOS mice induced by dehydroepiandrosterone (DHEA), including ovarian histopathology and function. Moreover, we found that the administration of MSCs significantly downregulated the expression of proinflammatory factors (TNF-*α*, IL-1*β*, and IFN-*γ*) and fibrosis-related genes (CTGF) in ovarian and uterus tissues and affected the systemic inflammatory response. The percentage of peripheral neutrophils, M1 macrophages, and B cells was significantly reduced, while M2 macrophages and regulatory T cells (Tregs) were increased in hUC-MSC-treated mice. In the spleen, the percentage of neutrophils, M1 macrophages, IFN-*γ*^+^CD19^+^B cell, IFN-*γ*^+^CD4^+^T cells (Th1), and IL-17^+^CD4^+^T cells (Th17) was significantly decreased in hUC-MSC-treated mice. These results suggested that hUC-MSC treatment could alleviate ovarian dysfunction by inhibiting ovarian local and systemic inflammatory responses.

## 1. Introduction

Polycystic ovary syndrome (PCOS) is one of the most common reproductive and endocrine diseases in women at a reproductive age, which is a common cause of women's menstrual irregularities and infertility [1]. The current diagnostic criteria of PCOS include ovulatory dysfunction, hyperandrogenism, and polycystic ovaries [2]. PCOS is frequently associated with insulin resistance, hyperinsulinemia, type 2 diabetes (T2D), obesity, hyperlipidemia, and increased cardiovascular risk, which is a heterogeneous syndrome, seriously endangering women's health [3]. Numerous studies showed that endocrinologic and metabolic abnormalities in PCOS may also have complex effects on the uterine tissue [4]; long-term PCOS increased the risk of endometrial hyperplasia and uterine cancer especially endometrial cancer (EC) [5]. In addition, despite its prevalence, the etiology and pathology of PCOS are still unknown. Besides genetic and environmental factors, immunity factor may also contribute to the development of PCOS. Women with PCOS had high levels of serum high-sensitivity C-reactive protein (hsCRP); serum inflammatory factors including tumor necrosis factor- (TNF-) *α*, interleukin- (IL-) 6, and interleukin- (IL-) 18; and serum autoantibodies including antinuclear (ANA), antithyroid, antispermatic, anti-SM, and antihistone [6, 7], which implied that PCOS is a chronic inflammatory and autoimmune disease. At present, there is no effective treatment for PCOS. Guidelines of PCOS in clinical practice were to change lifestyles, including exercise and dietary modification, or use drugs such as oral contraceptive pills (OCPs), metformin, and pioglitazone to relieve symptoms of hyperandrogenism and glucose tolerance [8].

Mesenchymal stem cells (MSCs) are multipotent adult stem cells with potent immunomodulatory properties. Studies reported that MSCs could inhibit T cell proliferation [9, 10], reduce the frequency of IFN-*γ*-producing T helper 1 (Th1) cells [11], and downregulate cytotoxic T cell- (CTL-) mediated cytotoxicity [12]. MSCs also inhibit B cell proliferation and maturation and decrease antibody (IgG, IgM) generation [13]. And for innate immune cells, it had been reported that MSCs could inhibit the maturation of monocytes, impair antigen-presenting function including dendritic cells and macrophages [14], decrease mast cell degranulation [15], and dampen the respiratory burst of neutrophils [16]. Because of its immunomodulatory properties, MSCs were promising to be a new treatment for severe refractory autoimmune diseases, such as rheumatoid arthritis (RA), systemic lupus erythematosus (SLE), atopic dermatitis (AD), systemic sclerosis, and type I diabetes (T1D) [17–19]. Human umbilical cord-derived MSCs (hUC-MSCs) have been reported to apply in both lupus mice and refractory SLE patients, which have significant improvement in disease activity and reduction in autoantibody levels [20, 21].

In this study, we assessed the therapeutic effects of hUC-MSCs on dehydroepiandrosterone- (DHEA-) induced PCOS mice. Results showed that hUC-MSC treatment significantly improved ovarian and uterine pathological changes of PCOS mice, including the polycystic ovaries, disordered estrous cycle, congested uterine, and edema and tissue fibrosis by inhibiting local and systemic inflammatory responses.

## 2. Materials and Methods

### 2.1. Animals

Female C57/BL6 mice (3 weeks old) were purchased from the Department of Animal Research, Xiangya Medical College, Central South University, China. Mice were housed in a pathogen-free facility with a 12-hour light-dark cycle. Food and tap water were allowed ad libitum. Animal surgery protocols were approved by the Animal Care and Use Committee of the Central South University.

### 2.2. Establishment of the PCOS Model and MSC Treatment

**Figure 1:**
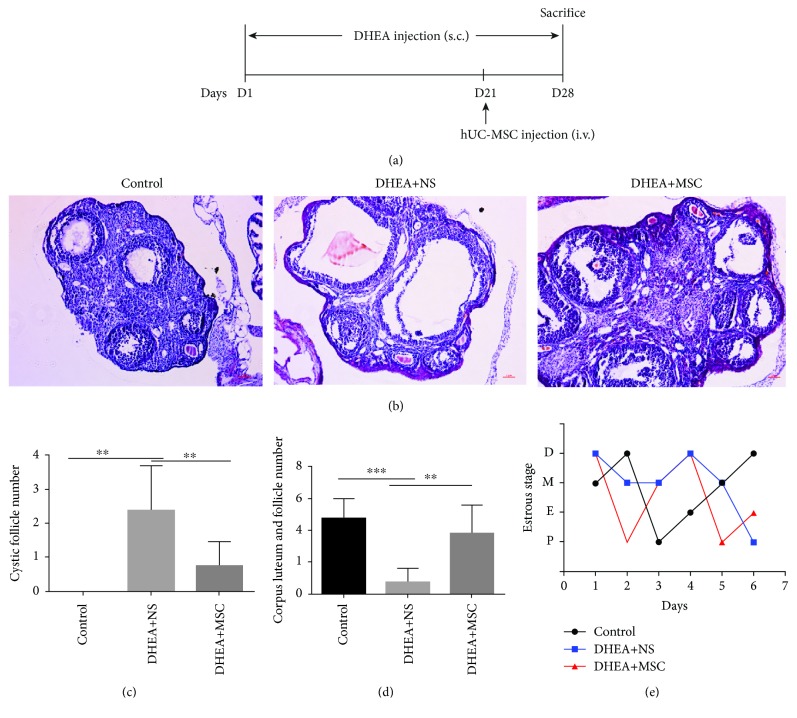
hUC-MSC treatment improves ovarian pathological changes and dysfunction in PCOS mice (a) Schematic of the experimental model. (b) Representative hematoxylin and eosin (H&E) staining of ovary section. Scale bars (50 *μ*m). (c) Numbers of cystic follicles in ovarian tissue. (d) Numbers of corpus luteum and mature follicles in ovarian tissue. (e) Representative results of estrous cycling. P: proestrus stage; E: estrus stage; M: metestrus stage; D: diestrus stage. Values are expressed as the means ± SEM. *n* = 8 per group. ^∗^*P* < 0.05 and ^∗∗^*P* < 0.01.

PCOS in mice was induced with DHEA (Sigma-Aldrich) as previously described [22]. Briefly, female prepuberal (21-day-old) C57BL/6 mice that received daily subcutaneous injections of DHEA (6 mg/100 g body weight, dissolved in 0.1 ml sesame oil [Acros]) for 21 days were classified as the DHEA group, while mice injected with sesame oil for an equivalent length of time as the control group. After the model of PCOS mice was established (the 21^st^ day of DHEA injection), hUC-MSCs (2 × 10^6^ passage 4 hUC-MSCs suspended in 0.2 ml normal saline) were transplanted to PCOS mice through the tail vein injection on day 21 and these mice were classified as the DHEA+MSC group, while those with an infusion of 0.2 ml normal saline alone were referred as the DHEA+NS group. To prevent ovarian self-recovery, we continued DHEA subcutaneous injection after hUC-MSC treatment for 7 days. After 7 days of infusing hUC-MSCs (the 28^th^ day of DHEA injection), all treated mice were sacrificed for further analysis, as shown in Figure 1(a). hUC-MSCs in this study were obtained from the National Engineering and Research Center of Human Stem Cell, and their isolation, culture, and identification were described in the previous study [23].

### 2.3. Identification of Estrous Cycle Stage

The estrous cycle of mice was assessed by vaginal cytology during six consecutive days from the 3^rd^ day after hUC-MSC treatment to the day of sacrifice. Samples were then treated with Giemsa stain (Baso, China) for 3 minutes, and consequently, cell morphology and estrous cycles were examined under an optical microscope (Nikon, Japan).

### 2.4. Histological Analysis

Ovarian and uterine tissues were fixed in 4% paraformaldehyde overnight and then embedded in paraffin. Sections of 5 *μ*m thickness were stained with hematoxylin and eosin (Baso, China), and then images were taken by a microscope (Nikon, Japan).

### 2.5. Flow Cytometry

Cells were labeled with antibodies purchased from BioLegend against mouse APC-CD19 (clone 6D5), FITC-CD3 (clone 17A2), APC-CD4 (clone GK1.5), PE-CD8 (clone 53-6.7), FITC-CD25 (clone PC61), PerCP-CD45 (clone 30-F11), PE-F4/80 (clone BM8), FITC-CD86 (clone GL-1), PE-CD11c (clone N418), PE-CD206 (clone C068C2), APC-Ly6G (clone 1A8), and PE-CD11b (clone M1/70). For intracellular cytokine staining, cells were cultured in the presence of 50 ng/ml phorbol 12-myristate 13-acetate (Sigma-Aldrich), 1 *μ*g/ml ionomycin (Sigma-Aldrich), and 1 *μ*g/ml monensin (BioLegend) for 5 hours. Cell surface CD3, CD4, and CD19 staining was performed first, and then, cells were fixed, permeabilized with Cytofix/Cytoperm and Perm/Wash solutions (Becton Dickinson), and subsequently stained with mouse PE-IFN-*γ* (clone XMG1.2), PE-IL-4 (clone 11B11), and PE-IL-17A (clone TC11-18H10.1). Data were acquired using a fluorescence-activated cell sorting (FACS) Aria I cytometer (Becton Dickinson) and analyzed using FACS Diva software (BD Biosciences).

### 2.6. Quantitative Real-Time PCR Analysis

Total RNA was prepared from frozen mouse ovarian and uterine tissues using TRIzol (Invitrogen, America), and cDNA was synthesized using a reverse transcription kit (Roche). Quantitative real-time PCR containing the SYBR Premix EX Taq™. cDNA and specific gene primers were run on the Roche LightCycler 480 II system (Roche). The relative expressions of each gene were determined and normalized to the expression of housekeeping gene glyceraldehyde 3-phosphate dehydrogenase (GAPDH) and calculated using the 2^−*ΔΔ*CT^ method. Gene primers are listed as follows: GAPDH—forward 5′-GCACCGTCAAGGCTGAGAAC-3′ and reverse 5′-TGGTGAAG ACGCCAGTGGA-3′; CTGF—forward 5′-CATTAAGAAGGGCAAAAAGTGC-3′ and reverse 5′-CACACCCCACAGAACTTAGCC-3′; F4/80—forward 5′-CCAGGAGTGGCTTTTGTCTC-3′ and reverse 5′-CTGGATGTGCTGGAGGGTAT-3′; IFN-*γ*—forward 5′-GCATCTTGGCTTTGCAGCT-3′ and reverse 5′-CCTTTTTCGCCTTGCTGTTG-3′; TNF-*α*—forward 5′-CTGAACTTCGGGGTGATCGG-3′ and reverse 5′-GGCTTGTCACTCGAATTTTGAGA-3′; IL-1*β*—forward 5′-GCAACTGTTCCTGAACT CAACT-3′ and reverse 5′-ATCTTTTGGGGTCCGTCAACT-3′; and IL-10—forward 5′-GAGATGCCTTCAGCAGAGTGAAGA-3′ and reverse 5′-AGTTCACATGCGCCTTGATGT C-3′.

### 2.7. Statistical Analysis

Data are presented as the means ± standard errors of the means (SEMs). Statistical analyses were performed using SPSS 22.0 and GraphPad Prism 6 statistical software. The statistical significance of differences between two groups was determined using two-tailed unpaired Student's *t*-tests. Differences among multiple groups were compared using one-way analyses of variance (ANOVAs) followed by Newman-Keuls analysis (^∗^*P* < 0.05, ^∗∗^*P* < 0.01).

## 3. Results

### 3.1. hUC-MSC Treatment Improves Ovarian Pathological Changes and Dysfunction in PCOS Mice

Polycystic ovarian, chronic oligo/anovulation, and irregular menstruation are the characteristics of PCOS. As shown in Figures 1(b)–1(e), the number of fluid-filled cystic follicles and the thickness of the theca cell layer were significantly increased, and the numbers of corpora lutea and dominant follicle were significantly decreased in DHEA+NS mice compared with control. In addition, DHEA+NS mice showed abnormal estrous cycling. These results indicated that DHEA induced the formation of PCOS in mice. However, the administration of MSC significantly decreased the number of cystic follicles and increased the number of mature follicles and corpus luteum and recovered the regular estrous cycle. The structure of the ovaries in MSC-treated mice was also comparable to that of those in the control group. These results indicated that MSC treatment could efficiency improve ovarian pathological changes and recovered ovulation in DHEA-induced PCOS mice.

### 3.2. hUC-MSC Treatment Improves Uterine Pathological Changes in PCOS Mice

**Figure 2:**
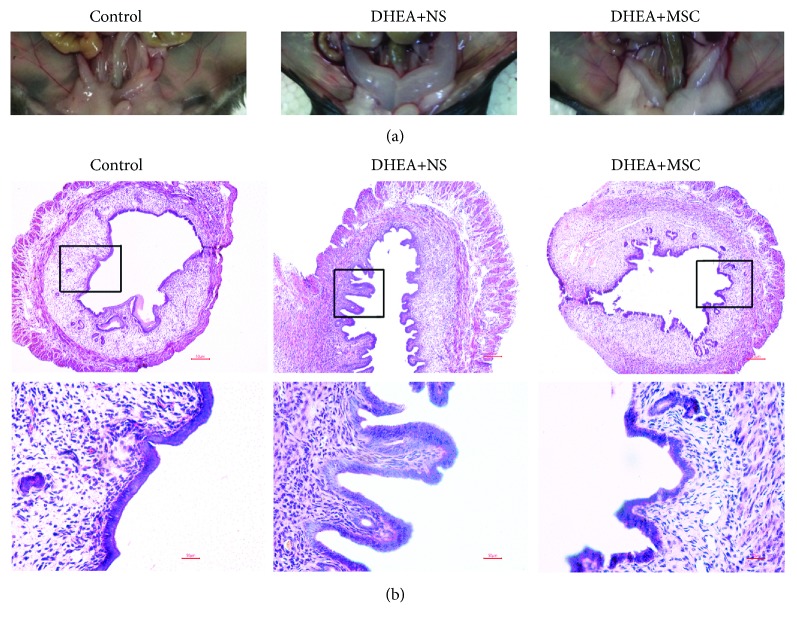
hUC-MSC treatment improves uterine pathological changes in PCOS mice. (a) Morphology of uterine tissue. (b) Representative hematoxylin and eosin (H&E) staining of uterus sections. Scale bars: 50 *μ*m.

Besides ovarian dysfunction, a woman with PCOS was frequently accompanied by endometrial disorders [4, 24]. In this study, we examined the changes of the uterine tissue in PCOS mice. Results showed that the uterine tissue in DHEA+NS mice was seriously congested and edema and the uterine canal was full of hydrocele (Figure 2(a)), indicating that the uterine tissue was in an inflammatory state. H&E staining also showed that DHEA+NS mice had abnormal thickening endometrial epithelium and increased uterine luminal diameter. However, MSC-treated mice showed normal uterine morphology and structure characterized by thinner endometrial epithelium and normal uterine luminal diameter similar to control (Figure 2(b)). These results indicated that hUC-MSC treatment could effectively improve uterus pathological changes in DHEA-induced PCOS mice.

### 3.3. hUC-MSC Treatment Alleviates Ovarian and Uterine Local Inflammatory Response and Tissue Fibrosis in PCOS Mice

**Figure 3:**
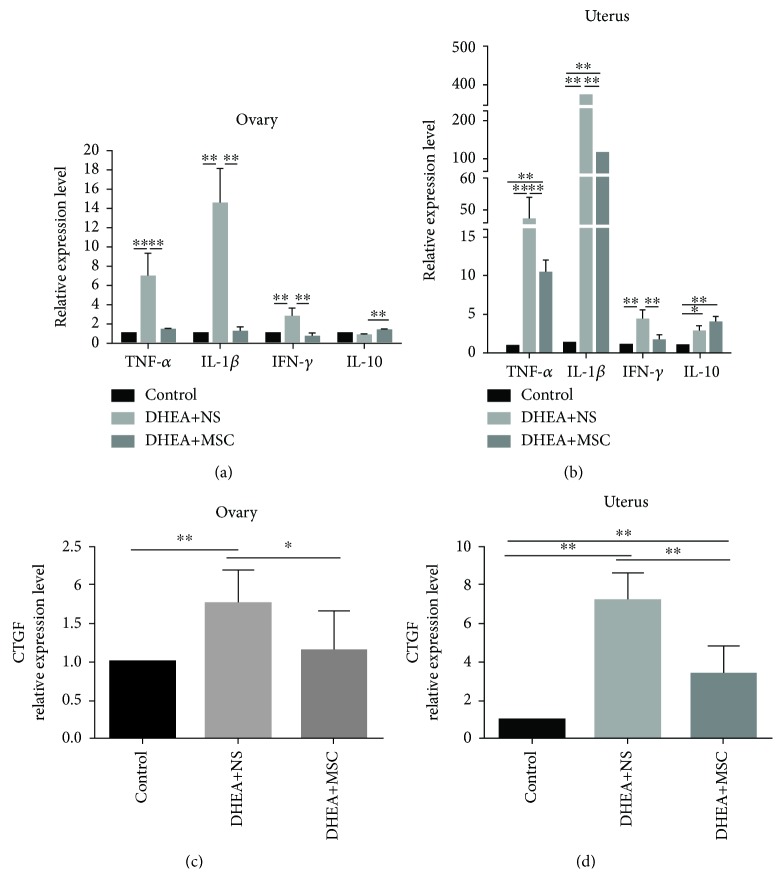
hUC-MSC treatment alleviates ovarian and uterine local inflammatory response and tissue fibrosis in PCOS mice. (a, b) Quantitative RT-PCR analysis of the expression of proinflammatory factors (TFN-*α*, IL-1*β*, and IFN-*γ*) and anti-inflammatory factor (IL-10) in the ovaries and uterus. (c, d) Quantitative RT-PCR analysis of the expression of connective tissue growth factor (CTGF) in the ovaries and uterus. Values are expressed as the means ± SEM. *n* = 8 per group. ^∗^*P* < 0.05 and ^∗∗^*P* < 0.01.

Chronic inflammation and hyperfibrosis had been occurring in a woman with PCOS [25, 26]. We examined the expression of the inflammatory factors (TNF-*α*, IL-1*β*, IFN-*γ*, and IL-10) and fibrosis factors (connective tissue growth factor (CTGF)) in mouse ovarian and uterine tissues. Results showed that the expression levels of the proinflammatory factors (TNF-*α*, IL-1*β*, and IFN-*γ*) in ovarian tissues of DHEA+NS mice were significantly increased compared with the control and the expression levels in MSC-treated mice were significantly decreased, and it is comparable to control (Figure 3(a)). Similar results were found in the uterine tissue (Figure 3(b)). However, the expression of anti-inflammatory IL-10 was elevated in the ovaries in MSC-treated mice, but the expression of IL-10 in uterine tissues showed a significant increase in both DHAE+NS and MSC-treated mice. We also found that the ovarian and uterine tissues in DHEA+NS mice expressed high levels of CTGF compared with control mice and, in the administration of MSC, significantly inhibited the expression of CTGF (Figures 3(c) and 3(d)). These results indicated that MSC treatment could alleviate ovarian and uterine local inflammatory response and tissue fibrosis by reducing proinflammatory factors (TNF-*α*, IL-1*β*, and IFN-*γ*) expression and increasing anti-inflammatory factor (IL-10) expression.

### 3.4. hUC-MSC Treatment Affects Innate Immunity Cell Compartments in PCOS Mice

**Figure 4:**
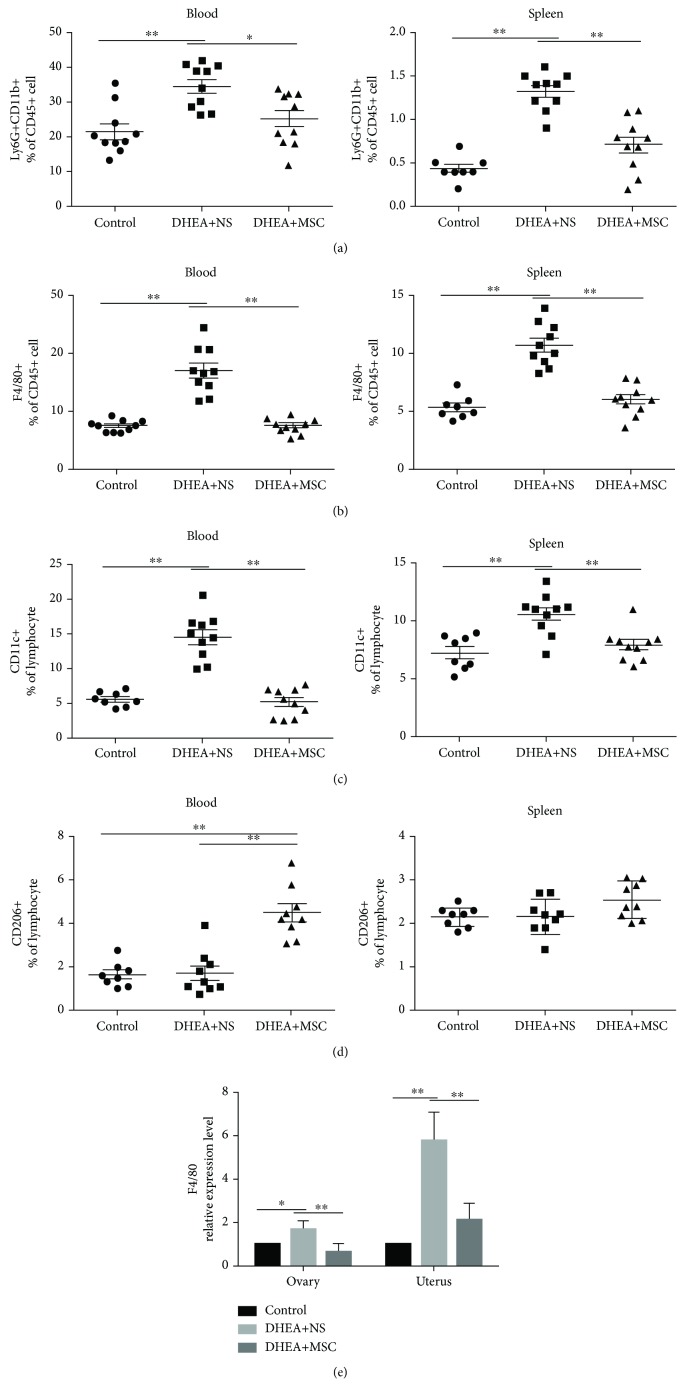
hUC-MSC treatment affects innate immunity cell compartments in PCOS mice. Flow cytometry results of innate immunity cells in the peripheral blood and spleen. (a) The percentage of peripheral and splenic neutrophils (Ly6G^+^CD11b^+^). (b) The percentage of peripheral and splenic macrophages (F4/80^+^). (c) The percentage of peripheral and splenic M1 macrophages (CD11c^+^). (d) The percentage of peripheral and splenic M2 macrophages (CD206^+^). (e) Quantitative RT-PCR analysis of the expression of F4/80 in the ovaries and uterus of mice. *n* = 8-10 per group. Values are expressed as the means ± SEM. ^∗^*P* < 0.05 and ^∗∗^*P* < 0.01.

Because MSCs have the immunomodulatory properties which could affect various immune cells, we examined the change of immune cells in DHEA-treated mice with and without MSC treatment. Firstly, we analyzed the change of innate immune cells including neutrophils and macrophages. Results showed that DHEA+NS mice had high percentages of the peripheral and splenic neutrophils (Ly6G^+^CD11b^+^) and macrophages (F4/80^+^) compared with the control, while neutrophils and macrophages in the MSC-treated group were comparable to those in control mice (Figures 4(a) and 4(b)).

We further analyzed macrophage subtypes M1 and M2 by labeling CD11c and CD206. Results showed that the percentage of the peripheral and splenic M1 macrophages (CD11c^+^) was significantly elevated in DHEA+NS mice (Figure 4(c)), while M2 macrophages (CD206^+^) did not show significant changes compared with the control mice (Figure 4(d)), indicating that the increased macrophages in DHEA+NS mice were mainly M1 macrophages, not M2 macrophages. The administration of MSC significantly reduced the percentage of peripheral and splenic M1 macrophages and increased the percentage of peripheral M2 macrophages, while splenic M2 macrophages did not change compared with DHEA+NS mice (Figures 4(c) and 4(d)). These results indicated that MSCs could modulate macrophage polarization, reducing the percentage of M1 macrophages while increasing M2 macrophages.

We also analyzed the expression of F4/80 in local ovarian and uterine tissues in PCOS mice. Results showed that the expression level of F4/80 in ovarian and uterus tissues in DHEA+NS mice was significantly higher than that in DHEA+MSC and the control mice (Figure 4(e)), suggesting that MSC treatment could decrease macrophage infiltration into both ovarian and uterine tissues.

### 3.5. hUC-MSC Treatment Affects T and B Cell Compartments in PCOS Mice

**Figure 5:**
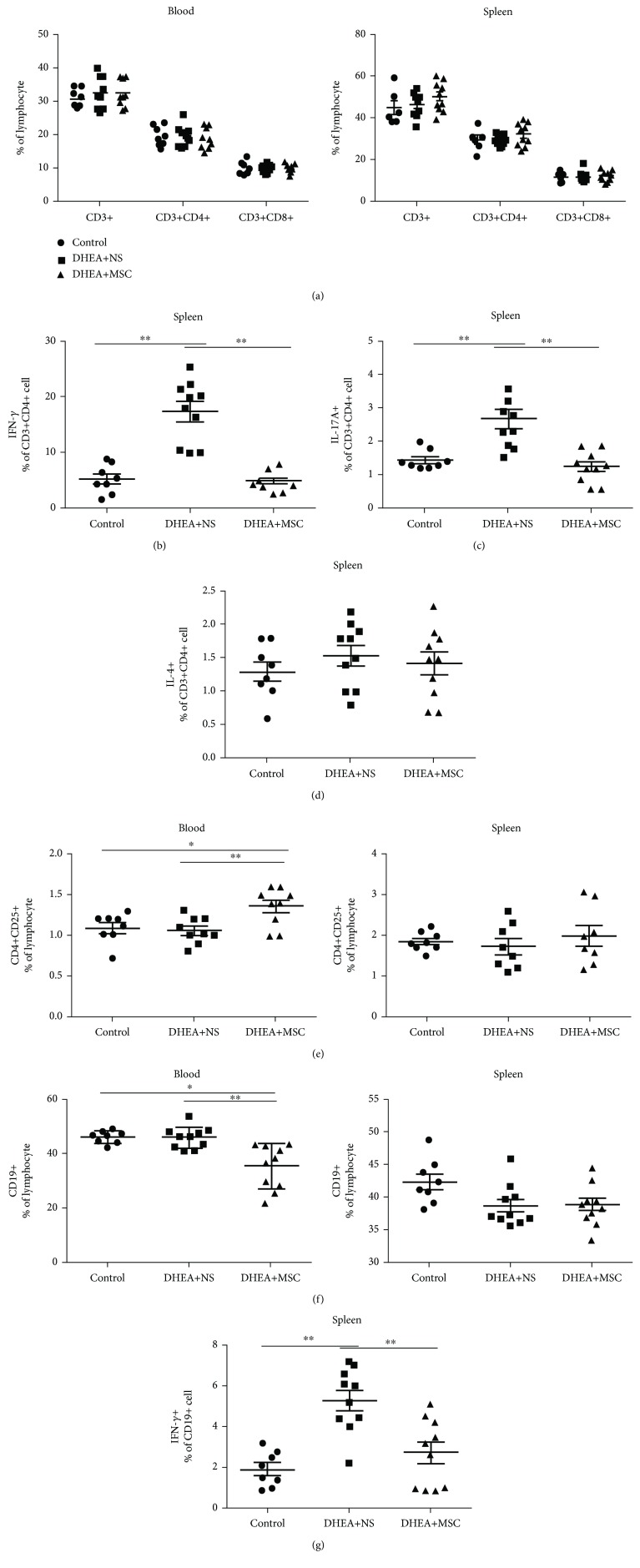
hUC-MSC treatment affects T and B cell compartments in PCOS mice. Flow cytometry results of T and B cells in the peripheral blood and spleen. (a) The percentage of peripheral and splenic T cells (CD3^+^), T helper cells (CD3^+^CD4^+^), and cytotoxic T cells (CD3^+^CD8^+^). (b) The percentage of splenic IFN-*γ*^+^T helper cells (Th1). (c) The percentage of splenic IL-17^+^T helper cells (Th17). (d) The percentage of splenic IL-4^+^T helper cells (Th2). (e) The percentage of peripheral and splenic Tregs (CD4^+^CD25^+^). (f) The percentage of peripheral and splenic B cells (CD19^+^). (g) The percentage of splenic IFN-*γ*^+^CD19^+^B cells. *n* = 8-10 per group. Values are expressed as the means ± SEM. ^∗^*P* < 0.05 and ^∗∗^*P* < 0.01.

Next, we evaluated the change of adaptive immunity cells including T and B cells in DHEA-induced mice with or without MSC treatment. Results showed that the percentage of peripheral and splenic T cells (CD3^+^), T helper cells (CD3^+^CD4^+^), and cytotoxic T cells (CD3^+^CD8^+^) was not significantly different among the groups (Figure 5(a)). We further analyzed the change of T helper cell subsets including Th1, Th2, Th17, and regular T cells (Tregs). Results showed that splenic IFN-*γ*^+^T helper cells (Th1) and IL-17^+^T helper cells (Th17) were significantly increased in DHEA+NS mice. The administration of MSC could significantly inhibit the differentiation of Th1/Th17; the percentages of Th1 and Th17 were comparable with the control group (Figures 5(b) and 5(c)). IL-4^+^T helper cells (Th2) did not show a statistical difference among the three groups (Figure 5(d)). MSC also increased the percentage of peripheral Tregs (CD4^+^CD25^+^) compared with other groups (Figure 5(e)). These results indicated that in DHEA-induced mice, hUC-MSCs could inhibit Th1 and Th17 differentiation and promote Treg differentiation.

MSC could inhibit B cell proliferation. In our study, the administration of MSC obviously decreased the percentage of peripheral B cells (CD19^+^) compared with DHEA+NS and control mice, while it did not show the same effects in the spleen (Figure 5(f)). The percentage of splenic IFN-*γ*^+^CD19^+^B cells in DHEA+NS mice was significantly increased, while the administration of MSCs could obviously decrease the percentage of IFN-*γ*^+^CD19^+^B (Figure 5(g)). These results indicated that MSC treatment could efficiently reduce the percentages of peripheral CD19^+^B cells and splenic IFN-*γ*^+^CD19^+^B cells.

## 4. Discussion

Chronic low-grade inflammation played an important role in the pathogenesis of PCOS [27]. Women with PCOS had increasing serum C-reactive protein (CRP) and proinflammatory cytokine level including TNF-*α*, IL-6, and IL-18 [28–30]. Ovarian follicular fluid from PCOS patients had an increasing number of activated T cells and white blood cells [31, 32], and peripheral blood neutrophils and Th17 cell proliferation were also elevated [33].

The present study, for the first time, clarified the therapeutic effect and the mechanism of MSCs on PCOS mice. Results demonstrated that hUC-MSC treatment could effectively alleviate pathological changes and improve ovarian function of PCOS mice, such as ovarian polycystic morphology, oligoovulation, disordered estrous cycle, uterus hyperemia and edema, and tissue fibrosis.

The capacity of MSC for multilineage differentiation as well as immunomodulation has meant that MSCs are highly versatile for a wide range of therapeutic application. A number of animal model and translational studies have reported that MSC could home to sites of injury and/or inflammation and participate in tissue repair by cell replacement, secreting cytokines, and regulating immune response [22, 23, 34]. The use of MSCs for in immune/inflammation-related diseases appears to yield more efficacy than for cell replacement/tissue regeneration. A study of the acute renal failure mouse model showed that MSC administration could increase the recovery of renal function through the inhibition of production of proinflammatory cytokines, such as IL-1*β*, TNF-*α*, and IFN-*γ* [35]. The anti-inflammatory activity of MSCs was also shown in a model of lung fibrosis. MSCs inhibited the effects of IL-1*α* producing T cells and TNF-*α* producing macrophages through the release of IL-1 receptor antagonist (IL-1RA) [36]. In addition, MSCs also had beneficial effects by increasing IL-10 production from lung monocytes and macrophages in a sepsis mouse model [37]. IL-1*β*, TNF-*α*, and IFN-*γ* were all proinflammatory factors, which could activate immune cells including neutrophils and lymphocytes, participating in the inflammation of the body. And IL-10 was an anti-inflammatory factor, which had been reported to inhibit rolling, adhesion, and transepithelial migration of neutrophils [38], further decreasing organ damage due to neutrophil invasion and relieving the inflammation of the body.

In this study, we used DHEA to induce a PCOS mouse model and found that the percentages of M1 macrophages and neutrophils were increased in both the peripheral blood and spleen of DHEA-induced mice and the percentages of splenic IFN-*γ*^+^T helper cells (Th1), IL-17A^+^T helper cells (Th17), and IFN-*γ*^+^CD19^+^B cell were also increased, and the expression of proinflammatory factors (TNF-*α*, IL-1*β*, and IFN-*γ*) in the ovarian and uterine tissues was increased. These results indicated that the immune system of DHEA-induced mice was activated which was similar to a PCOS patient.

Our results showed that hUC-MSC transplantation could inhibit the expression of proinflammatory factors (IFN-*γ*, TNF-*α*, and IL-1*β*) and increase the expression of anti-inflammatory factor (IL-10) in local ovarian and uterine tissues in DHEA-induced mice, suggesting that hUC-MSCs could induce a proinflammatory state shift to an anti-inflammatory state, further alleviating the pathological changes and function on local ovarian and uterine tissue in PCOS mice. However, DHEA+NS mice also showed an increased expression of IL-10 in this study. This increase might be due to the compensation, to stop the inflammation in mice.

It had been reported that MSC transplantation had notable curative effectiveness in mouse models of macrophage hyperactivity, such as sepsis and zymosan-induced peritonitis. Studies also reported the capacity for MSCs to shift the balance of M1/M2 macrophage polarization both in vitro and in vivo [39–41]. Classically activated M1 macrophages play a central role in host defense by producing proinflammatory cytokines such as IL-1*β* and TNF-*α*, while alternatively activating M2 macrophages producing anti-inflammatory cytokines and growth factors, contributing to inflammation suppression, wound healing, and tissue regeneration [42]. In a study of T2D mouse model, MSC infusion could improve glucose homeostasis and restored islet function by modulated macrophage polarization. MSCs could inhibit classical M1 activation and convert macrophages into an anti-inflammatory M2 phenotype [43]. This property was also mentioned in a rheumatoid arthritis (RA) mouse model. In this study, the results showed that the percentage of macrophages was reduced in both the peripheral blood and spleen of MSC-treated mice compared with DHEA-induced mice. hUC-MSCs could alleviate inflammatory response in PCOS mice by affecting macrophage polarization, reducing peripheral M1 macrophages and increasing M2 macrophages. Our study also showed that hUC-MSCs could inhibit the percentage of the peripheral and splenic neutrophils that might be due to the increase of IL-10 expression as previously mentioned.

MSCs could inhibit T cell proliferation and decrease IFN-*γ* production, which inhibited proinflammatory (IFN-*γ*-producing) state both in vitro [11] and in vivo [17]. It has been reported that MSCs exert its therapeutic potential in experimental arthritis by inhibiting Th1 and Th17 responses and inducing regulatory T cell response [44]. Consistent with these reports, we found that hUC-MSCs could significantly reduce the percentage of splenic IFN-*γ*^+^Th1 cells and IL-17A^+^Th17 cells and increase the percentage of peripheral Tregs in DHEA-induced PCOS mice.

MSCs could inhibit B cell proliferation and terminal differentiation. In a study of atopic dermatitis (AD) mouse model, MSCs were applied to suppress the allergic responses by inhibiting the proliferation and maturation of B cells [15]. MSCs could also inhibit the production of TNF-*α* and IFN-*γ* by B cells when B cells were cocultured with MSCs [10, 13]. In this study, our results showed that there was a significant reduction in the percentage of peripheral B cells (CD19^+^) and splenic IFN-*γ*^+^CD19^+^B cells in MSC-treated mice compared with DHEA+NS mice. Thus, hUC-MSCs could alleviate inflammatory response by inhibiting Th1/Th17 cells and B cells producing proinflammatory factors and inducing Treg differentiation in DHEA-induced PCOS mice.

In conclusion, this study demonstrated that hUC-MSC treatment could efficiently improve the pathological changes and function of PCOS by inhibiting local (ovaries and uterus) and systemic inflammatory responses in PCOS mice.

## Data Availability

The data used to support the findings of this study are included within the article.
